# The draft genome of *Spiraea crenata* L. (Rosaceae) – the first complete genome in tribe Spiraeeae

**DOI:** 10.1038/s41597-024-03046-0

**Published:** 2024-02-17

**Authors:** Levente Laczkó, Sándor Jordán, Szilárd Póliska, Hanna Viktória Rácz, Nikoletta Andrea Nagy, Attila Molnár V., Gábor Sramkó

**Affiliations:** 1 Department of Metagenomics, University of Debrecen, Debrecen, Hungary; 2 HUN-REN–UD Conservation Biology Research Group, University of Debrecen, Debrecen, Hungary; 3 Juhász-Nagy Pál Doctoral School, University of Debrecen, Debrecen, Hungary; 4 Department of Biochemistry and Molecular Biology, Faculty of Medicine, University of Debrecen, Debrecen, Hungary; 5 Department of Biotechnology and Microbiology, Faculty of Science and Technology, University of Debrecen, Debrecen, Hungary; 6 Department of Evolutionary Zoology and Human Biology, Faculty of Science and Technology, University of Debrecen, Debrecen, Hungary; 7 HUN-REN–UD Behavioural Ecology Research Group, University of Debrecen, Debrecen, Hungary; 8 Evolutionary Genomics Research Group, Department of Botany, Faculty of Science and Technology, University of Debrecen, Debrecen, Hungary

**Keywords:** Evolutionary biology, Evolutionary genetics, Plant genetics

## Abstract

*Spiraea crenata* L. is a deciduous shrub distributed across the Eurasian steppe zone. The species is of cultural and horticultural importance and occurs in scattered populations throughout its westernmost range. Currently, there is no genomic information on the tribe of Spiraeeae. Therefore we sequenced and assembled the whole genome of *S. crenata* using second- and third-generation sequencing and a hybrid assembly approach to expand genomic resources for conservation and support research on this horticulturally important lineage. In addition to the organellar genomes (the plastome and the mitochondrion), we present the first draft genome of the species with an estimated size of 220 Mbp, an N50 value of 7.7 Mbp, and a BUSCO score of 96.0%. Being the first complete genome in tribe Spiraeeae, this may not only be the first step in the genomic study of a rare plant but also a contribution to genomic resources supporting the study of biodiversity and evolutionary history of Rosaceae.

## Background & Summary

**Fig. 1:**
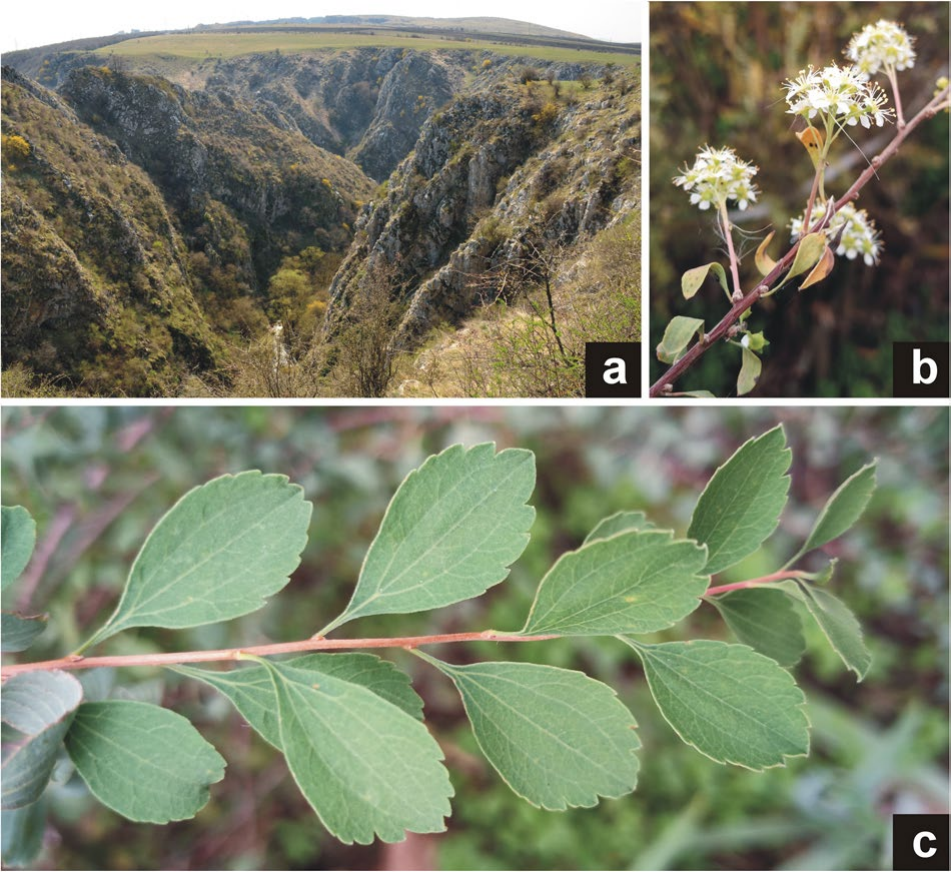
The original habitat in Tureni, Romania (**a**), inflorescence (**b**), and foliage (**c**) of the *Spiraea crenata* specimen used in the current work (photographs taken by G.S.).

*Spiraea crenata* L. (Rosaceae) (Fig. 1), colloquially called scalloped spiraea, is a deciduous shrub characteristic of the Eurasian true steppe zone^1^. The distribution range of this species extends across the zone from southeastern Europe on the west, with fragmented populations in the Iberian Peninsula, to the Altai Mountains on the east^2^. In the westernmost regions of its range, the species can be considered as being of special conservation interest as a relict of steppe flora (see Palou *et al*.^3^ and Molnár *et al*.^4^). *S. crenata* occurs on stony, calcareous slopes (Fig. 1a) and in steppe shrublands often on sand and can grow up to 1 m tall. The leaves are oblong-elliptic, 2–4 cm long, about 1 cm wide with three characteristic, approximately parallel main veins in the middle (Fig. 1c). The 6 to 8 mm wide, white-petaled flowers grow on stalks (Fig. 1b) that are 5 to 10 mm long. The stamens are longer than the petals and form an inflorescence about 2 cm wide^5^.

Like many species of the Rosaceae, the genus *Spiraea* is of great horticultural importance^6^. The value of *S. crenata* in gardening is demonstrated by specimens recently discovered in cemeteries in Hungary possibly transplanted to the graves for ornamental use as the last remnants of the former natural flora of the region^4^. Nevertheless, the species is considered to be extinct in several European countries in the western part of its former range (e.g. Denmark, Bulgaria, Hungary) due to large scale loss of its habitat Some of the last European remnant populations can be found in rift valleys where the impact of intensive grazing is less significant (Fig. 1a). However, the recent discovery of individuals in urban environments^4,5^ presents an opportunity for re-introduction if the indigenous nature of the plants can be proven using conservation genomic tools. Above this, the area-wide conservation genomics of this characteristic steppe shrub can help us better understand the evolutionary significance of the European peripheral populations.

Most wild species belonging to the horticulturally and agronomically important Rosaceae have not been characterized at the genomic level to date. Their available genomic resources are dominated by cultivated species. The tribe Spiraeeae is no exception: the few publicly available genomic resources are limited to plastomes^7^. Moreover, this tribe belongs to the subfamily Amygdaloideae, which comprises a large number of domesticated and economically important species (e.g. apple, pear, almond). The phylogenetic position of the tribe is uncertain, but it most probably represents one of the basal clades^8^. This uncertainty is partly due to a whole-genome duplication event and allopolyploidy during the early evolution of the subfamily. Recent phylogenetic data point to Spiraeeae as the source of this hybridization^9^, and the genome of *S. crenata* can be an important source for studying the role of this tribe in the evolution of this important subfamily.

**Fig. 2:**
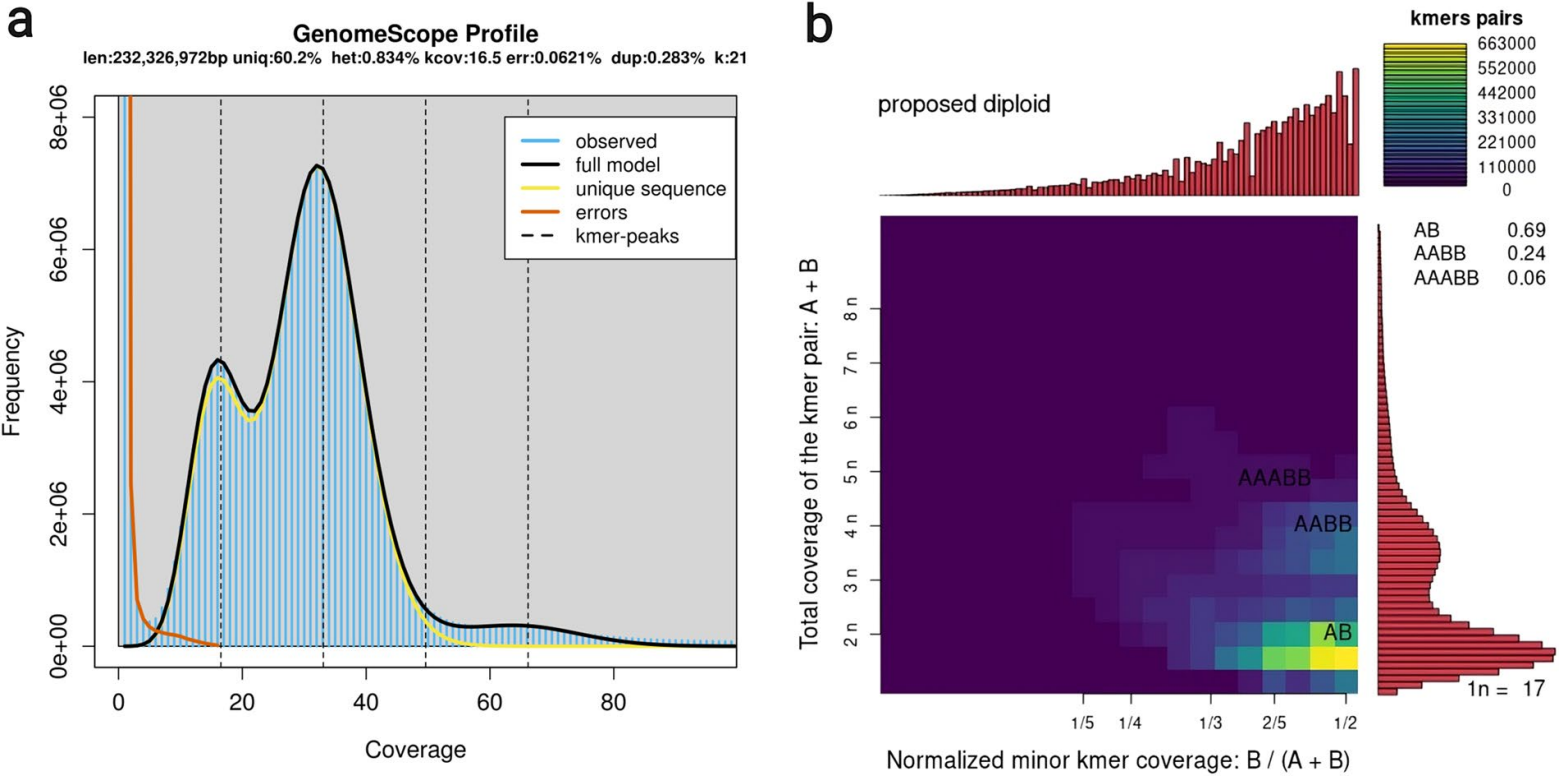
K-mer coverage statistics of the short-read dataset. Panel ‘a’ shows estimated genome size and complexity as output by GenomeScope, and panel ‘b’ shows ploidy assessment using Smudgeplot.

**Fig. 3:**
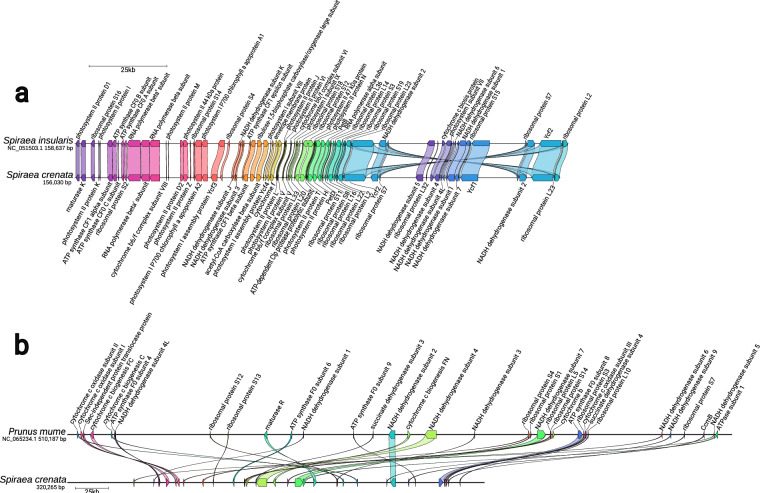
Structural comparison of the de novo reconstructed plastome (**a**) and mitochondrial genome (**b**) with the reference used for organellar read identification. The colors of the links refer to the identity of the genes and their width corresponds to the size of the genes. The arrows indicate the orientation of the genes. The horizontal black lines representing the organellar genomes are proportional to the genome size. We have edited the figure output by Clinker in Inkscape to improve readability.

Here we report the assembled genome of *S. crenata*, by which we would like to contribute to the understanding of the origin and genomic characteristics of the Spiraeeae. For *de novo* assembly, we used a combination of MGIseq short and Oxford Nanopore MinION long reads. We present the complete chloroplast and mitochondrial genomes of the species as well as the draft nuclear genome. We estimate the size of the genome, which is diploid, to be approximately 220 Mbp (Fig. 2). The assembly of the organellar genomes of the sequenced sample appears to be complete (Fig. 3). The polished and decontaminated nuclear genome assembly has a total size of 217.7 megabase pairs (Mbp), an N50 of 7.7 Mbp (Table 1), and a BUSCO score of 96.0% (Table 2). We have predicted 35,264 protein-coding genes, of which 39.21% were involved in biological processes (BP), 19.79% in cell component formation (CC), and 40.98% in molecular functions (MF) (Fig. 5). Our phylogenetic analysis placed *S. crenata* as sister to the Maleae and Amygdaleae and showed similar gene density to *Prunus* sp. (Fig. 7). Reconstruction of this genome is not only the first step in the genomic study of a rare plant that contributes to genomic resources for conservation, but may also promote progress in deciphering the evolutionary relationships within Rosaceae and in clarifying the taxonomic classification of the genus Spiraea based on genomic information^7^.

**Table 1:** Contiguity assessment of the *Spiraea crenata* assemblies created based on QUAST^31^ statistics during the process of genome assembly.

Assembly Feature	nextDenovo	MaSuRCA	Merged (MaSuRCA + nextDenovo)	Merged pseudohaploid	Merged and reduced
# contigs (> = 0 bp)	115	139	94	93	89
# contigs (> = 1k bp)	115	139	94	93	89
# contigs (> = 5k bp)	115	139	94	93	89
# contigs (> = 10k bp)	115	138	94	93	89
# contigs (> = 25k bp)	115	138	94	93	89
# contigs (> = 50k bp)	113	137	93	92	88
Total length (> = 0 bp)	212,791,066	202,127,248	218,618,502	218,457,710	217,666,113
Total length (> = 1k bp)	212,791,066	202,127,248	218,618,502	218,457,710	217,666,113
Total length (> = 5k bp)	212,791,066	202,127,248	218,618,502	218,457,710	217,666,113
Total length (> = 10k bp)	212,791,066	202,122,159	218,618,502	218,457,710	217,666,113
Total length (> = 25k bp)	212,791,066	202,122,159	218,618,502	218,457,710	217,666,113
Total length (> = 50k bp)	212,711,542	202,094,899	218,574,431	218,413,639	217,622,567
# contigs	115	139	94	93	89
Largest contig	14,311,357	15,493,676	14,302,248	14,302,248	14,289,189
Total length	212,791,066	202,127,248	218,618,502	218,457,710	217,666,113
GC (%)	39.28	39.22	39.24	39.23	39.21
N50	5,658,611	2,226,269	7,704,760	7,704,760	7,686,064
N90	1,216,363	709,943	1,503,707	1,503,707	1,599,821
auN	6,274,732.6	4,243,811.9	7,681,326.7	7,686,862.1	7,704,330.3
L50	12	19	11	11	11
L90	42	78	32	32	31
# N’s per 100 kbp	0	14.54	0.51	0.51	0.46

**Table 2:** Genome completeness estimated by BUSCO^32^ using the eudicots_odb10 ortholog dataset at different stages of the genome assembly process.

BUSCO genes	nextDenovo	MaSuRCA	Merged (MaSuRCA + nextDenovo)	Merged pseudohaploid	Merged and reduced
Complete (%)	2,179 (93.68)	2,175 (93.51)	2,233 (96.00)	2,233 (96.00)	2,233 (96.00)
Single copy (%)	2,117 (91.01)	2,049 (88.09)	2,085 (86.94)	2,085 (86.94)	2,085 (86.94)
Duplicated (%)	162 (6.96)	126 (5.42)	148 (6.36)	148 (6.36)	148 (6.36)
Fragmented (%)	13 (0.56)	19 (0.82)	17 (0.73)	17 (0.73)	17 (0.73)
Missing (%)	34 (1.46)	132 (5.67)	76 (3.27)	76 (3.27)	76 (3.27)
Total number of BUSCOs	2,326	2,326	2,326	2,326	2,326

The table shows the number of BUSCOs in each category along with their percentage of the whole ortholog dataset between parentheses.

## Methods

### Sample collection and sequencing

Plant material was collected from a garden plant originating from the gorge Cheile Tureniului, also known as Túri-hasadék (near the settlement Tureni, CJ, Romania) (latitude: 46.61302°, longitude: 23.709557°; altitude: 529 m a.s.l.). It was cultivated in a common garden patch at the Botanical Gardens of the University of Debrecen (Hungary). We isolated total genomic DNA from 500 mg of freshly collected leaf tissue sample according to the modified CTAB protocol of Doyle & Doyle (1987). We ground the freshly collected leaves with sterile SiO_2_ and PVPP under liquid nitrogen in a pre-chilled (−80 °C) mortar and then added 1,600 μl of prewarmed (65 °C) extraction buffer (2% CTAB, 0.5% 2-mercaproetanol, 0.04% PVP) to the pulverized tissue. We then divided the homogenizate into two equal parts and incubated the tubes at 65 °C for 45 minutes with constant shaking (260 RPM) and removed the debris by centrifugation at 10,000 RPM for 2 minutes. We added 320 μg RNAseA (Macherey-Nagel, Dueren, Germany) to the lysate and incubated it for 10 minutes at room temperature. The lysate was then washed twice with an equal volume of chloroform:isoamyl alcohol 24:1, and the tubes were gently inverted for 5 minutes. The precipitate was removed by centrifugation at 10,000 RPM for 3 minutes, and then 0.1 V ammonium acetate (7.5 M) and 2 V isopropanol were added at room temperature to precipitate the DNA. After incubation on ice for 10 min, the DNA pellets were collected by centrifuging the tubes at 10,000 RPM for 3 min, and the supernatant was poured off. We washed the pellets twice with 70% ethanol at room temperature and, after drying at room temperature, resuspended the genomic DNA in 100 μl 10 mM Tris-HCl (pH = 8.0). We removed any remaining contaminants by adding 0.8 V AMPure XP beads (Beckman Coulter, Brea, CA, USA) to the isolates, following the manufacturer’s recommendations for DNA purification, and resuspending the genomic DNA in 50 μl 10 mM Tris-HCl (pH = 8.0) buffer. We verified the high molecular weight of the isolates by loading 50 ng of DNA onto a 1% agarose gel, ensuring purity with a NanoDrop 2000 (Thermo Fisher Scientific, Waltham, MA, USA), and measuring dsDNA concentration with a Qubit 3.0 fluorometer (Thermo Fisher Scientific, Waltham, MA, USA).

We randomly sheared 500 ng of genomic DNA using the Bioruptor Plus (Diagenode, Liège, Belgium) and adenylated the 3′ ends of the 200–500 base pair (bp) long fragments after size selection using magnetic beads (SPRI SureSelect Kit, Beckman Coulter, Brea, CA, USA). We then prepared the sequencing library using MGIEasy Universal DNA Library Prep Set v1.0 (MGI Tech Co., Ltd., Shenzhen, China) according to the manufacturer’s instructions. We sequenced the library on a DNBSEQ-G400RS platform (FCL PE150). To enrich long fragments in the Oxford Nanopore sequencing library, we used the standard Short Read Eliminator (SRE) kit from Circulomics (Baltimore, MD, USA). Before library preparation, we rechecked the molecular weight, purity, and concentration of the isolate. We prepared the sequencing library with 1,000 ng of DNA following the recommendations of the Genomic DNA by Ligation Sequencing Kit (SQK-LSK110, Oxford Nanopore Technologies, Oxford, UK). We loaded a total of 600 ng of the prepared library onto an R10.3 MinION flow cell (Oxford Nanopore Technologies). We generated raw data for 72 hours using a MinION mk1b instrument, washed the flow cell after 36 hours using the Flow Cell Wash Kit (EXP -WSH003), and reloaded the library after refilling the flow cell. Raw MinION sequencing data were basecalled with Guppy 5.0.11 (Oxford Nanopore Technologies) using the Super High Accuracy basecalling model (dna_r10.3_450bps_sup) to achieve the highest possible accuracy per read.

### Read quality filtering and preprocessing

As the first step of short read preprocessing, we assessed the quality of raw DNBSEQ reads using FastQC 0.11.9^10^ and then filtered them using fastp 0.20.1^11^. Fastp used a sliding window of 5 base pairs (bp) to trim sequences at both the 5′ and 3′ ends with a mean Phred quality score of less than 15 (--cut_front 20 --cut_tail 20 --cut_window_size 5 --cut_mean_quality 15) and an unqualified percent cutoff of 50%. We enabled base correction for overlapping regions and turned on adapter detection for PE sequencing. After filtering, we retained 15.69 gigabase pairs (Gbp) of sequencing data (104,315,474 reads) from 16.25 Gbp of raw data. We then used Bloocoo 1.0.6^12^, a read error corrector that operates based on the *k*-mer spectrum with default options to reduce the sequencing error rate, and re-evaluated the result using FastQC. We observed unbalanced base composition in the first 10 bps and also at the 3′ end of the reads (about the last 100 bps), even after read error correction. To reduce sequencing errors in whole genome reconstructions, we trimmed the first 10 and last 40 bps of reads with cutadapt 2.8^13^, resulting in 10.46 Gbp of quality-filtered sequencing data. We then estimated the *k*-mer frequency spectrum of the trimmed dataset using KMC 3.1.1^14^, setting the minimum frequency of *k*-mers to be considered to 1 (-ci1), the maximum frequency to 10,000 (-cs10000), and the *k*-mer length to 21 (-k21). To assess genome size, we analyzed the resulting frequency histogram with GenomeScope^15^ and estimated the ploidy of the sequenced sample with smudgeplot 0.2.3^15^ using the same *k*-mer histogram as GenomeScope. Smudgeplot used a lower and upper *k*-mer coverage threshold set to 8 and 285, respectively, with smudgeplot.py cutoff. We visualized the results with smudgeplot.py plot. Genomescope estimated a size of 230 Mbp with a unique *k*-mer content of 60.2% and 0.834% heterozygosity (Fig. 2a), and smudgeplot showed that the sequenced sample was diploid (Fig. 2b).

We evaluated the MinION sequencing run using MinIONQC 1.4.2^16^ and then excluded the reads of the DNA control strand using NanoLyse 1.2.0^17^. We used NanoFilt 2.8.0^17^ to trim 50 bp of reads at both the 5′ and 3′ ends to ensure that all adaptor sequences were removed and to exclude reads with a mean quality of less than 7 or with a length of less than 500 bp. Finally, we evaluated and visualized the read quality metrics using NanoPlot 1.38.1^17^. After quality filtering, we retained 1,049,962 reads with a total base count of 9,42 Gbp and a read N50 of 18,633 bp.

### Genome assembly

Because plant cells contain more than one copy of organelles (i.e., the plastome and the mitochondrion)^18^, organellar genomes may well be overrepresented in sequencing datasets^19^. Identifying and then separately assembling nuclear and organellar reads could compensate for biases arising from unequal coverage of different genomic compartments^20^. To this end, we successively aligned both short and long reads to the reference chloroplast of *Spiraea insularis* (NC_051503.1) and the reference mitochondrion of *Prunus mume* (NC_065234.1), which were the most closely related taxa with available organellar reference genomes at that time. We used bwa 0.7.17^21^ to align the short reads and minimap 2.17-r941^22^ to align the long reads to the organellar reference sequences. Although there are numerous tools for short-read assembly of organellar genomes (e.g. GetOrganelle^23^, NovoPlasty^24^), we are not aware of any *de novo* assembler explicitly designed for assembling organellar genomes using a hybrid assembly approach. To take advantage of both short and long reads, we used Unicycler 0.5.0^25^ – developed for bacterial genome assembly – for short-read-first assembly of the chloroplast and mitochondrial genomes. Since the mitochondrial assembly using Unicycler resulted in multiple genomic fragments even when allowing a higher misassembly rate (--mode bold) to compensate for the distant relatedness of the original reference (NC_065234.1), we first iteratively aligned the short and long reads to the newly generated assemblies and assembled those identified as mitochondrial *de novo*. Although Unicycler was still assembling multiple fragments after five iterations, the number of reads in the mitochondrial dataset could not be further increased. Therefore, we used MaSuRCA 4.0.5^26^ – a general purpose genome assembler – for hybrid assembly of the iteratively aligned reads. All assemblies were successively polished using racon 1.4.22^27^, medaka 1.72 (https://github.com/nanoporetech/medaka) using the r103_sup_g507 model, and pilon 1.23^28^. Organellar genomes were annotated by annotation transfer using Liftoff 1.6.3^29^, for which we specified sequence annotation of reference genomes used to identify organellar reads prior to assembly. The structure of the *de novo* assembled organellar genomes was assessed by comparison with the corresponding reference sequences using clinker 0.0.27^30^. The 156,030 bp long plastome appears circular and shows a typical quadripartite structure consisting of a large single copy (LSC) and small single copy regions (SSC), as well as two inverted repeats, which contained the coding genes *rpl*2, *rpl*23, *ycf*2, *ndh*B, *rps*7, and *rps*12 (Fig. 3a) identically to the reference plastome of *S. insularis*. The mitochondrion is 320,265 bp long and has larger rearrangements compared with the reference mitochondrion of *P. mume*, but all genes of the reference could still be identified with high similarity (Fig. 3b).

To exclude organellar reads from the assembly of the nuclear genome, we aligned the quality-filtered and error-corrected sequencing data to the *de novo* assembled plastid and mitochondrial genomes as described above and removed reads with an alignment block length greater than 95% of the read length. We then used the remaining short and long reads to reconstruct the nuclear genome and performed two assemblies. Assembly contiguity and completeness were checked using QUAST 5.2.0^31^ and BUSCO 5.2.2^32^ at all stages of the assembly process. BUSCO searches relied on the odb10 databases for Eukaryota, Viridiplantae, Embryophyta, and Eudicots.

For the first assembly, we used nextDenovo 2.5.0 to assemble the long reads^33^, setting the ‘input_type’ to raw, the ‘read_type’ to ont, and the expected ‘genome_size’ to 230 Mbp, as estimated by GenomeScope. This assembly had a total size of 212.8 Mbp (Table 1) and consisted of 115 contigs with an N50 value of 5.57 Mbp and a percentage of complete BUSCOs of 93.68%. In the second assembly, we used MaSuRCA 4.0.5^26^ to use both short and long reads in the same assembly step and obtained an assembly with a total size of 202.1 Mbp, which consisted of 139 contigs with an N50 value of 2.22 Mbp (Table 1) and in which 93.51% of BUSCO genes were complete (Table 2). We merged the two assemblies with quickmerge 0.3^34^, using the primary assembly of MaSuRCA as a hybrid and the contigs reconstructed by nextDenovo as a self-assembly, resulting in a more contiguous draft genome than either of the two primary assemblies, consisting of 94 contigs with a total size of 218.6 Mbp, an N50 value of 7.7 Mbp, and a complete BUSCO ratio of 96.0%. We polished the assemblies before and after merging in the same manner as for the organellar genomes.

To compensate for the effect of high heterozigosity of plant genomes (as also estimated by GenomeScope), we removed partially resolved duplicated fragments by pseudohaploid (https://github.com/schatzlab/pseudohaploid) using create_pseudohaploid.sh, after which the genome was polished again, which reduced the number of contigs to 93 but had no effect on the N50 value of the contigs and the BUSCO value. Since the assembly still contained 6.36% duplicated BUSCOs, we used redundans 0.11^35^ with --nogapclosing and --noscaffolding options enabled. We set both the minimum overlap and identity to 0.95 (--identity 0.95 --overlap 0.95), as any parameter combination with lower values reduced the proportion of complete BUSCOs. After genome reduction, the assembly consisted of 89 contigs with a total size of 217.7 Mbp and an N50 of 7,686,064 bp with BUSCO scores unchanged. The assembly was polished with racon, medaka and pilon before and after duplicated fragment detection.

**Fig. 4:**
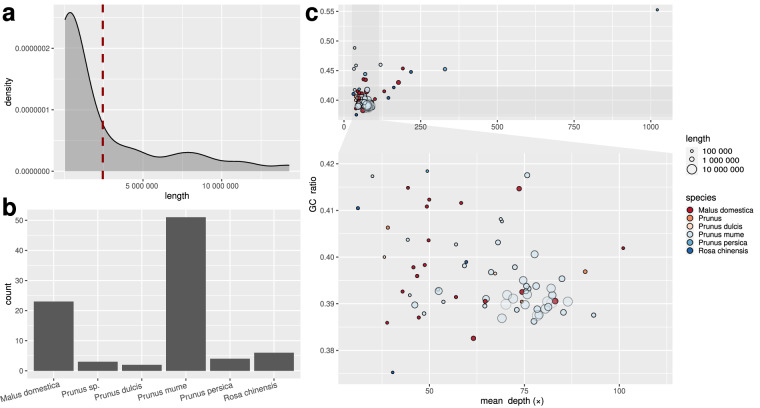
Contamination control of the *Spiraea crenata* genome showing the length distribution of contigs, with mean length shown as a red dashed line (**a**), abundance of species identified with kraken2 using contigs as input (**b**), and mean read depth plotted against GC ratio (**c**). On panel c, the size of the circles is proportional to the contig length, and the colors represent the different species classified by kraken2.

We screened contaminant sequences by running kraken 2.1.2^36^ with the k2_pluspfp database (version 6/7/2022; https://genome-idx.s3.amazonaws.com/kraken/k2_pluspfp_20220607.tar.gz), then estimated the length and GC ratio of contigs with bedtools nuc 2.26.0^37^ and the mean read depth using alignments of both short- and long-read sequencing datasets with samtools coverage 1.10^38^. The GC ratio ranged from 0.37 to 0.55, the length of the contigs ranged from 43.4 kbp to 14.29 Mbp (Fig. 4a,c), and the mean read depth varied from 31.16 × to 1,021.33 × (Fig. 4c). Kraken2 classified all contigs as members of the Rosaceae family (Fig. 4b); therefore, we did not identify any contaminants and retained all contigs for subsequent analyses.

### Genome annotation

**Fig. 5:**
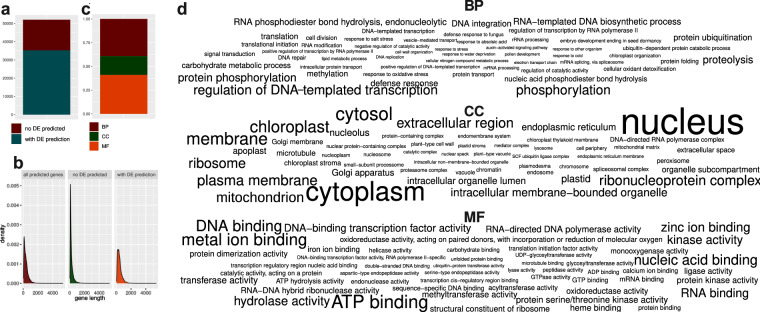
Functional annotation of the genome of *Spiraea crenata*. The figure shows the number (**a**) and length (**b**) of all *ab initio* and homology-based gene predictions and the number of genes that could be functionally annotated with free text descriptions (DE). The ratio of GO terms (BP, CC, MF) is shown as a stacked bar graph (**c**) and the 50 most frequent functions for each of these three categories are shown as a word clouds (**d**).

We soft-masked repeat regions in the genome using Red 2.0^39^, identifying 199,722 repeat regions with a minimum length of 13 bp and a maximum length of 100,861 bp (mean = 378.77 bp). We then annotated rRNAs with barnnap 0.9 (https://github.com/tseemann/barrnap), tRNAs with ARAGORN 1.2.38^40^, and predicted coding gene sequences with the BRAKER 2.1.6^41,42^ pipeline. For gene prediction, we used Augustus 3.3.3^43^ for *ab initio* and GeneMark- ES Suite 4.69_lic^44^ for homology-based prediction. We used OrthoDB v10 plant protein sequences^45^ (https://v100.orthodb.org/download/odb10_plants_fasta.tar.gz) as evidence for homology-based prediction. We created a consensus sequence set from the *ab initio* and homology-based predictions using CD-HIT 4.7^46^ with command line parameters -c 1 -G 0 -aL 1.0 -aS 1.0. The unique coding sequences were functionally annotated using the PANNZER web server^47^ (http://ekhidna2.biocenter.helsinki.fi/sanspanz/). In this way, we identified 52,009 putative genes, of which 35,264 (67%) could be functionally annotated (Fig. 5a). Longer genes could be assigned to a functional description (DE) more frequently than shorter ones (Fig. 5b). 39.21% of the functionally annotated genes played a role in biological processes (BP), with RNA-mediated DNA biosynthesis processes, proteolysis, protein phosphorylation, phosphorylation, and regulation of DNA-mediated transcription being the most common functions. The GO term “cellular component” (CC) corresponded to 19.79% of genes, and the most common functions included formation of the structure of the chloroplast, membrane, cytosol, cytoplasm, and nucleus. 40.98% of the functional annotations showed involvement in molecular functions (MF), and zinc ion binding, nucleic acid binding, DNA binding, metal ion binding, and ATP binding were the most common processes (Fig. 5c,d).

**Fig. 6:**
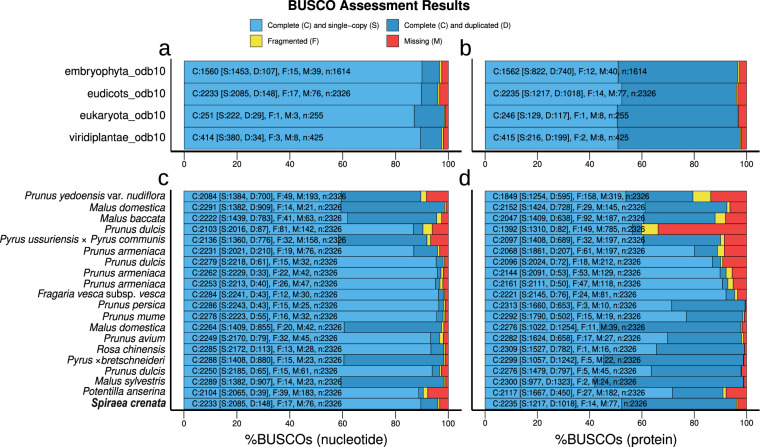
BUSCO analysis of the newly assembled genome of *Spiraea crenata* (**a**) and the annotated proteome (**b**) using different ortholog datasets, and the comparison of the completeness of the de novo assembled genome with the available genomes (**c**) and proteomes (**d**) of Rosaceae using the Eudicots v10 ortholog database (eudicots_odb10).

To assess the quality of the final assembly, BUSCO 5.2.2^32^ was used to estimate the completeness of the whole genome and proteome (i.e., functionally annotated protein-coding genes) using the Embryophyta, Eudicot, Eukaryota, and Viridiplantae odb10 databases. In a next step, the completeness of the genome and proteome of *S. crenata* was assessed by comparing BUSCO results with the genomes of other Rosaceae for which functional genome annotation was available. The ortholog database eudicots_odb10 was used for these BUSCO searches. The completeness of *S. crenata* was > 96% in all cases (Fig. 6a), and the proportion of duplicates (6.0–11.4%) varied according to the ratio of complete genes. Similar completeness was observed for the annotated proteome, although a much higher duplication rate of 43.8% to 45.8% was observed (Fig. 6b). This phenomenon was also observed for most publicly available Rosaceae genomes (Fig. 6c,d). The completeness of assembly (96%) and proteome (96.1%) of *S. crenata* were comparable to those of the species included in the analysis (89.6–98.5% and 59.8–99.5%, respectively) (Fig. 6c,d).

### Orthology assessment

**Fig. 7:**
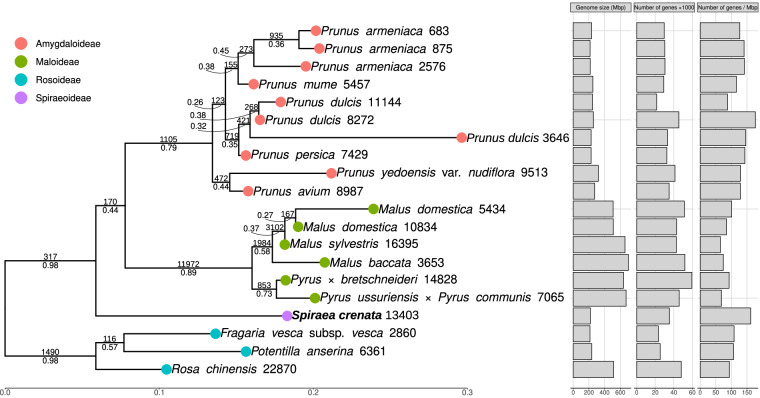
Species tree of Rosaceae proteomes reconstructed with OrthoFinder. aLRT support values are indicated below, and the number of gene duplications above the branches of the phylogenetic tree and the number of gene duplications at the terminal branches are indicated next to the species names. Genome size, total number of functionally annotated genes, and gene density are shown as bar graphs next to the species tree.

We then assessed gene orthology and performed phylogenetic inference from the available, well-annotated proteomes of Rosaceae (Table 3), supplemented with the proteome of *S. crenata*, using OrthoFinder 2.4.0^48^ with default settings. Of the total 756,065 genes, 731,251 (96.72%) could be assigned to one of the 39,406 orthogroups, of which 33,829 (85.84%) were present in at least two species. In the reconstruction of the species tree, Rosoideae were identified as the root and *S. crenata* was placed as sister to the Maleae and Amygdaleae (Fig. 7). Many internal branches received poor statistical support, which is most likely due to the known hybridisation ability of the group (e.g. Hodel *et al*.^49^), which could reduce phylogenetic support values^50^. Nonetheless, the tribes of Amygdaloideae were clearly identified as distinct units, and the reconstructed topology was concordant with the phylogenetic hypothesis presented by Xiang *et al*.^8^, but notably inconsistent with that based on whole plastomes (Zhang *et al*.)^51^. Within the ingroup, the genome size of *S. crenata* (217.66 Mbp) was most similar to members of Amygdaleae (215.24–319.21 Mbp) and also had a similar number of genes (35,264 and 21,564–45,581). Consequently, the gene density of *S. crenata* (162.01) also appeared to be most similar to members of Amygdaleae (87.61–176.90) and showed the second highest gene density after an accession of *Prunus dulcis* (GCA_021292205.2), whose accessions had both the lowest and highest gene densities (Fig. 7; see Table 3). The number of gene duplications on the terminal branch leading to *S. crenata* (13,403), which was the only representative of Spiraeeae included in this analysis, was comparable to the number of duplications on the branch leading to the Maleae and fit well within the range of duplications observed on terminal branches (683–22,870).

**Table 3:** Publicly available genome sequences used for the assessment of gene orthology and completeness of the final *Spiraea crenata* assembly.

Species	NCBI Assembly accession number	Subfamily	Tribe	Genome size (Mbp)	Number of genes as available in NCBI Genome database
*Rosa chinensis*	GCF_002994745.2^57^	Rosoideae	Roseae	515.119	48,188
*Potentilla anserina*	GCF_933775445.1^58^	Rosoideae	Potentilleae	236.974	25,318
*Fragaria vesca* subsp. *vesca*	GCF_000184155.1^59^	Rosoideae	Potentilleae	214.373	23,319
*Malus baccata*	GCA_006547085.1^60^	Amygdaloideae	Maleae	674.412	45,900
*Malus sylvestris*	GCF_916048215.2^61^	Amygdaloideae	Maleae	641.527	59,561
*Malus domestica*	GCF_002114115.1^62^	Amygdaloideae	Maleae	703.358	52,036
*Malus domestica*	GCA_004115385.1^63^	Amygdaloideae	Maleae	660.463	42,841
*Pyrus ussuriensis* × *Pyrus communis*	GCA_008932095.1^64^	Amygdaloideae	Maleae	510.637	43,120
*Pyrus* × *bretschneideri*	GCF_019419815.1^65^	Amygdaloideae	Maleae	509.113	51,345
*Prunus avium*	GCF_002207925.1^66^	Amygdaloideae	Amygdaleae	272.362	35,009
*Prunus yedoensis* var. *nudiflora*	GCA_002966975.2^67^	Amygdaloideae	Amygdaleae	319.21	41,294
*Prunus persica*	GCF_000346465.2^68^	Amygdaloideae	Amygdaleae	227.569	32,595
*Prunus dulcis*	GCF_902201215.1^69^	Amygdaloideae	Amygdaleae	227.757	33,326
*Prunus dulcis*	GCA_021292205.2^70^	Amygdaloideae	Amygdaleae	257.659	45,581
*Prunus dulcis*	GCA_008632915.2^71^	Amygdaloideae	Amygdaleae	246.117	21,564
*Prunus armeniaca*	GCA_020424065.1^72^	Amygdaloideae	Amygdaleae	251.33	29,211
*Prunus armeniaca*	GCA_903112645.1^73^	Amygdaloideae	Amygdaleae	215.952	30,573
*Prunus armeniaca*	GCA_903114435.1^74^	Amygdaloideae	Amygdaleae	215.24	30,315
*Prunus mume*	GCF_000346735.1^75^	Amygdaloideae	Amygdaleae	234.03	29,705

## Data Records

We deposited all data described in this study in the NCBI database under BioProject PRJNA1003507. The raw data belonging to BioSample SAMN36892215 can be found in the Sequence Read Archive (SRA) database under accessions SRX21302384^52^ and SRX21302383^53^, whereas the *S. crenata* genome assembly can be found in the Assembly database under accession GCA_033992175^54^. This Whole Genome Shotgun project has been deposited at GenBank under the accession JAVBHV000000000. The version described in this paper is version JAVBHV010000000.1. The structural and functional annotation of the assembly is made public in the Zenodo data repository^55^ under 10.5281/zenodo.8226512.

## Technical Validation

We thoroughly filtered short reads using fastp and error-corrected them using Bloocoo prior to any downstream analysis, including assessment of genome size, ploidy, and genome assembly. Similarly, we filtered long reads with NanoFilt to remove low-quality reads, ensure a relatively low error rate, and increase assembly contiguity. We compared the organellar genomes to the most closely related organellar reference genome using clinker to validate their structure and annotation. We polished all assemblies with Racon, medaka and pilon before and after each step in order to increase contiguity, and controlled the quality of the assemblies in terms of contiguity and completeness using QUAST and BUSCO. We ensured that the final assembly was free of contamination by checking the taxonomic classification, length, and GC ratio of the contigs. We ensured the validity of genome annotation by using *ab initio* and evidence-based predictions, checking the completeness of the proteome with BUSCO, and then assessing the number of functionally annotated genes and the number of gene duplications in a phylogenetic context. Using phylogenomic reconstruction we further assessed the accuracy of the genome of *Spiraea crenata*. Within the Amygdaloideae subfamily, where the understanding of evolutionary history is complicated by ancient hybridization and whole genome duplication^8^, our results were consistent with a previous phylogenetic hypothesis, and the gene count and CDS density were similar to the most closely related species. This confirms the accuracy of functional annotations and the completeness of the genome.

## Data Availability

We did not use any custom code in this study. The versions and parameters of the bioinformatic tools used in this study were described in the Methods section. If a parameter was used with other than its default value, this was stated above as well.
